# Incidence of Subclinical Hypothyroidism in Obese Adults and Its Metabolic Implications: A Prospective Cohort Study

**DOI:** 10.7759/cureus.95661

**Published:** 2025-10-29

**Authors:** Umer Jameel, Obaidullah Durrani, Ahmad Munib, Amanullah Khan

**Affiliations:** 1 Acute Medicine, Russells Hall Hospital, Dudley, GBR; 2 Emergency Medicine, EasyMed Medical Complex, Zafarwal, PAK; 3 Orthopaedics, Sargodha Medical College, Sargodha, PAK; 4 Internal Medicine, Shaikh Zayed Hospital, Lahore, PAK; 5 Medical and Health Research, University of the Punjab, Lahore, PAK; 6 Medical Research, University of Health Sciences, Lahore, Lahore, PAK

**Keywords:** hypothyroidism, insulin resistance, metabolic syndrome, obesity, thyroid function tests

## Abstract

Background: Subclinical hypothyroidism (SCH) is characterized by elevated thyroid-stimulating hormone (TSH) and normal free thyroxine (FT4) levels. It may also assume the status of comorbidity with obesity and may cause augmented dyslipidemia, insulin resistance, and cardiovascular disease. The aim of the current study was to estimate the prevalence of SCH and its association with the metabolic aberrations in obese adults.

Methods: In this prospective cohort study, the random sampling method was used to recruit 200 obese adults (body mass index (BMI) 30 kg/m^2^ and above) who were tested during six months in Shaikh Zayed Hospital, Lahore, Pakistan. The clinical and demographic data of the anthropometric measurements were also recorded. The laboratory tests were interpreted to identify serum TSH, FT4, fasting blood glucose, lipid profile, and insulin resistance calculated through the homeostatic model assessment of insulin resistance (HOMA-IR). SCH featured TSH.4.5 mIU/L with normal FT4. The data were analyzed by using the chi-square test, independent t-tests, and Pearson with p < 0.05.

Results: SCH was present in 41.0% (82/200) of obese adults. SCH patients had significantly greater BMI, fasting glucose, HOMA-IR, total cholesterol, and LDL-C (all p < 0.05) but lower levels of HDL-C (p = 0.004). TSH showed positive correlations with BMI (r = 0.32), HOMA-IR (r = 0.41), and lipid abnormalities and a negative correlation with HDL-C (r = -0.22, p = 0.046).

Conclusion: SCH was seen to be more common in obese adults and was strongly associated with poor metabolic phenotypes. Thyroid screening within the early years of obese patients would facilitate an appropriate early diagnosis and treatment process to mitigate cardiometabolic risk.

## Introduction

Subclinical hypothyroidism (SCH) is an increasing and recognized concern in global health [1]. This condition is clinically explained through the higher levels of thyroid-stimulating hormone (TSH) in the bloodstream, whereas levels of free thyroxine (fT4) remain within the normal range [1]. Although SCH rarely demonstrates overt symptoms, it is seen to be associated with cardiovascular and metabolic abnormalities, which, if left untreated, can lead to apparent hypothyroidism [2]. It is reported in studies that the incidence of SCH in the world varies around 4-10 %, and a higher incidence is observed in women and the elderly [3].

Dysendocrine is becoming a growing issue with respect to obesity, which is a major social health issue [4]. Some researchers also claim that patients with increased body mass indices (BMIs) have more chances of developing SCH, and this might be attributed to a maladjustment of the hypothalamic-pituitary-thyroid axis and leptin signalling disruption [5]. Moreover, SCH and obesity may result in the development of metabolism-related issues, such as insulin resistance, dyslipidemia, and hypertension [6]. The comorbidity of both risk factors predetermines the presence of a significant risk of cardiovascular disease and type 2 diabetes mellitus, which is of clinical significance [7]. More concern has been expressed regarding the prevalence of SCH in obese individuals because there is emerging evidence to suggest that obesity may not only predispose individuals to thyroid dysfunction but also augment its metabolic effect [8]. Longitudinal studies have suggested that the incidence rates among certain cohorts of people need to be quantified and dictate preventive actions.

The purpose of this study was to examine the SCH in obese patients and its connection to related metabolic dysfunctions. By investigating the etiology of thyroid function, obesity, and metabolic health associations and identifying patterns of their occurrence, this study suggested the findings that the development of targeted screening and preventive measures.

## Materials and methods

This prospective cohort study was carried out at an outpatient Endocrinology Department of Shaikh Zayed Hospital, a tertiary care unit affiliated with Punjab University, Lahore, between September 2023 and February 2024. The study was approved by the Institutional Review Board of Punjab University (Ref: 137/09/2023). Adult male and female obese patients attending regular follow-up visits were recruited through a random sampling method, with a total of 200 participants enrolled, followed by written informed consent taken from each participant. The sample size was estimated by using OpenEpi version 3.0.0 (released 2013, Atlanta, GA, USA), considering a 95% confidence level, 5% margin of error, and an anticipated incidence of 15% of SCH in obese populations [9].

The study included adult participants who were aged ≥30 years, BMI of ≥30 kg/m², were euthyroid at baseline (TSH ≤ 4.5 m IU/L, normal FT4), and completed both baseline and six-month follow-up assessments. These follow-ups included thyroid and metabolic profiling, such as fasting glucose, lipid profile, and Homeostatic Model Assessment of Insulin Resistance (HOMA-IR). Participants who had thyroid disorders before, recently used thyroid-altering drugs (amiodarone, lithium, glucocorticoids), had pregnancy or lactation, had incomplete data, or were lost to follow-up were excluded. Individuals with psychiatric or cognitive impairments that affected their compliance were also excluded.

The demographic variables (age, gender), anthropometric measurements (weight, height, BMI), blood pressure, and metabolic history comprised the baseline and follow-up evaluations. The thyroid and metabolic parameters were reassessed at the three- and six-month follow-up visits. After an overnight fast of eight to 12 hours, venous blood samples (5 mL) were collected in the morning (8-10 AM). A final analytical sample of 200 participants was obtained by excluding those who were lost to follow-up (n = 5; two because of relocation, three because they withdrew consent). In addition, incomplete lab results that resulted in missing data were not included in the analysis. The ADVIA Centraur XP chemiluminescent immunoassay system (Siemens, Germany) was used to measure serum TSH and FT4, and it was calibrated in accordance with the manufacturer's instructions.

Using enzymatic spectrophotometric assays on the Cobas c311 analyzer (Roche Diagnostics, Switzerland), the fasting glucose and lipid profile were evaluated in accordance with standard laboratory procedures. The formula for HOMA-IR was (fasting insulin (µU/mL) × fasting glucose (mg/dL)). Internal quality control procedures were used every day to standardize results and preserve reproducibility, and all assays were performed in multiple batches to guarantee reliability. IBM SPSS Statistics for Windows, Version 26.0 (IBM Corp., Armonk, NY) was used to analyze the data. Baseline characteristics were summarized using descriptive statistics (p-value < 0.05 significant). The associations between TSH levels and metabolic parameters were assessed using Pearson correlation analysis. Statistical significance was defined as a p-value <0.05.

## Results

The incidence and metabolic associations of SCH in individuals with obesity were examined. Due to relocation (n = 3) or consent withdrawal (n = 2), five participants (2.5%) were lost during the six-month follow-up period. In order to ensure minimal attrition bias, 200 participants were included in the final analysis. Metabolically disturbed patients (elevated BMI, dyslipidemia, and insulin resistance) experienced a very large number of cases of SCH when compared to non-metabolically disturbed patients. Table 1 illustrates clinical and demographic features of the research participants.

**Table 1:** Clinical and demographic features of the study population *Significance at p-value < 0.05

Parameter	Total (n = 200)	SCH present (n = 82)	SCH absent (n = 118)	Statistical test	Test value	p-value
Age (years) (Mean ± SD)	46.8 ± 10.5	47.6 ± 9.8	46.2 ± 11.1	t-test	0.92	0.358
Male gender, n (%)	112 (56.0%)	44 (53.7%)	68 (57.6%)	Chi-square	0.28	0.598
BMI (kg/m²) (Mean ± SD)	30.2 ± 4.7	31.5 ± 4.5	29.2 ± 4.6	t-test	3.28	0.001*
Duration of obesity (years)	7.8 ± 4.9	8.9 ± 5.0	7.0 ± 4.7	t-test	2.65	0.009*
Family history of thyroid disease, n (%)	54 (27.0%)	30 (36.6%)	24 (20.3%)	Chi-square	6.12	0.013*

The BMI and the period of obesity in patients with SCH were significantly greater compared to those without SCH (p = 0.001 and p = 0.009, respectively). Notably, SCH patients had a higher incidence of a family history of thyroid disease (30 (36.6%) vs. 24 (20.3%)), which raises the possibility of a genetic predisposition that could contribute to the development of SCH independently or in combination with obesity. There were no significant variations in age and gender. Table 2 summarizes metabolic abnormalities in patients with and without SCH.

**Table 2:** Metabolic profile in patients with and without subclinical hypothyroidism *Significance at p-value < 0.05

Variable	Total (n = 200)	SCH present (n = 82)	SCH absent (n = 118)	Statistical test	Test value	p-value
Fasting blood glucose (mg/dL) (Mean ± SD)	107.5 ± 22.4	113.2 ± 21.6	103.5 ± 22.5	t-test	2.92	0.004*
HOMA-IR (Mean ± SD)	2.9 ± 1.2	3.4 ± 1.3	2.6 ± 1.1	t-test	4.24	<0.001*
Total cholesterol (mg/dL) (Mean ± SD)	198.6 ± 38.1	209.8 ± 40.2	190.5 ± 35.9	t-test	3.41	0.001*
LDL-C (mg/dL) (Mean ± SD)	124.5 ± 32.0	133.6 ± 34.2	118.1 ± 29.8	t-test	3.29	0.001*
HDL-C (mg/dL) (Mean ± SD)	42.6 ± 9.5	40.1 ± 8.9	44.3 ± 9.7	t-test	–2.89	0.004*

Fasting glucose (113.2 ± 21.6 vs. 103.5 ± 22.5, p = 0.004), insulin resistance (3.4 ± 1.3 vs, 2.6 ± 1.1, p < 0.001 ), and unfavorable lipid profiles (increased total cholesterol (209.8 ± 40.2 vs. 190.5 ± 35.9, p = 0.001), LDL-C (133.6 ± 34.2 vs. 118.1 ± 29.8, p = 0.001) and reduced HDL-C (40.1 ± 8.9 vs. 44.3 ± 9.7, p = 0.004)) were significantly higher in patients with SCH as compared to those without SCH, respectively. Table 3 presents the correlation between the metabolic parameters and thyroid functioning (TSH levels) of the SCH patients.

**Table 3:** Correlation between thyroid stimulating hormone levels and metabolic parameters in subclinical hypothyroidism patients *Significance at p-value < 0.05.

Variable pair	Correlation coefficient (r)	Statistical test	Test value (t)	p-value
TSH vs. BMI	0.32	Pearson’s r	3.04	0.003*
TSH vs. HOMA-IR	0.41	Pearson’s r	4.12	<0.001*
TSH vs. total cholesterol	0.29	Pearson’s r	2.71	0.007*
TSH vs. LDL-C	0.27	Pearson’s r	2.48	0.014*
TSH vs. HDL-C	–0.22	Pearson’s r	–2.01	0.046*

**Figure 1:**
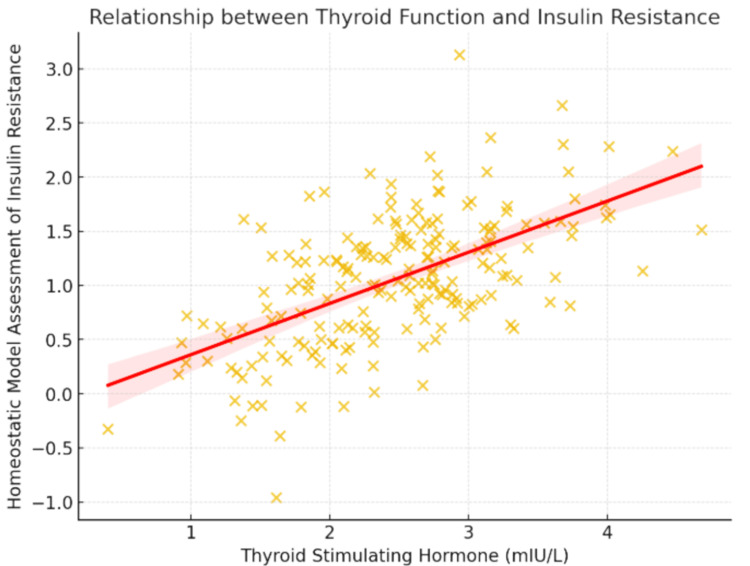
Scatter plot showing the positive correlation between TSH and HOMA-IR levels among the study participants Thyroid function and insulin resistance are strongly correlated, as evidenced by the positive correlation between thyroid-stimulating hormone (TSH) and homeostatic model assessment of insulin resistance (HOMA-IR) (r = 0.41, p < 0.001).

Metabolic parameters had a positive association with TSH levels and BMI (r = 0.32), insulin resistance (r = 0.41), and a negative association with HDL-C (r = -0.22). A scatter plot showing this relationship clearly shows the trend of increasing HOMA-IR with higher TSH levels, highlighting the finding's clinical significance, as shown in Figure 1.

## Discussion

This research determined the prevalence of SCH among the obese subjects and the connection between SCH and metabolic disturbances. The results indicated that SCH was found to be significantly higher in obese patients who had an increased metabolic risk profile. Particularly, SCH was well linked to BMI and family history of thyroid disorder. The relationship that was found between SCH and obesity in the study fell in agreement with the past statistics. The association between high body fat mass and BMI and high levels of TSH has been reported, demonstrating that excess adiposity may be a risk factor for hypothyroidism [10]. In a research, it was noted that the TSH of overweight and obese individuals was much higher compared to individuals who were of normal weight, which showed that adiposity affected thyroid regulation [11]. On the same note, other studies also proved that SCH can result in weight gain by lowering the basal metabolic rate, resulting in a vicious cycle of obesity and thyroid dysfunction [12].

It was also found that metabolic abnormalities like dyslipidemia and insulin resistance were more prevalent in patients with SCH. These findings were in line with the earlier results, which outlined that SCH had a positive correlation with total cholesterol and LDL-C level, which were also the risk factors of cardiovascular disease [13,14]. It was further noted that the insulin resistance of obese patients with SCH was more than that of the euthyroid patients, which demonstrated that thyroid malfunction was also a possible contributor to the deterioration of the metabolic syndrome [15]. Such effects are believed to be mediated by reduced expression of LDL receptors, disturbed lipid metabolism, and insulin resistance caused by deficiency of thyroid hormones [16].

Notably, the present study also found that there was a positive correlation between TSH with BMI, TSH with HOMA-IR, and TSH with atherogenic lipid markers. This supported the concept that thyroid abnormalities could enhance an obese person's risk of cardiometabolic disease. Though certain researchers had indicated that mild SCH might not be clinically relevant, there was growing evidence indicating that mild SCH was likely to contribute to metabolic risk profile exacerbation [17]. Although levothyroxine might help improve lipid disorders and reduce cardiovascular severity in certain individuals, the treatment of SCH in obese patients would remain controversial [18,19].

A major limitation of this research is that, as a six-month prospective cohort, it allows the evaluation of correlations but not the establishment of causation between metabolic abnormalities and SCH. Significant confounding variables that may have impacted the observed metabolic outcomes were not measured and not controlled, e.g., dietary patterns, physical activity, and other lifestyle patterns. Furthermore, the generalizability of the findings is limited due to the single-center design and moderate sample size of the study. The possibility of documenting chronic metabolic changes associated with SCH may also have been restricted by the relatively short follow-up period. Furthermore, certain factors that may further affect thyroid function were not measured, such as iodine intake, medication intake, and stress levels. However, these limitations indicate the need for longer-term, multicentre prospective studies of larger sample sizes, incorporating lifestyle and biochemical factors, to better define causal pathways and possible intervention strategies.

## Conclusions

Insulin resistance and hyperlipidemia were two adverse metabolic abnormalities that showed significant correlation with SCH, which was quite common among obese individuals. The excess of SCH prevalence in subjects that remained obese for a longer period of time suggests a cumulative effect of long-lasting adiposity on thyroid function. These results show positive relations between TSH, BMI, and important metabolic indicators that may suggest that thyroid dysfunction might worsen the cardiometabolic burden of obesity.

The study reinforces the importance of routine thyroid screening among obese people in order to promote the early detection and treatment of SCH. Nevertheless, as this study was observational and its results show correlations rather than cause and effect. To determine the causality and whether treatment of SCH in a timely way can ameliorate long-term metabolic and cardiovascular outcomes, more longitudinal and interventional research is needed.
